# Evaluation of real-time PCR of patient pleural effusion for diagnosis of tuberculosis

**DOI:** 10.1186/1756-0500-4-279

**Published:** 2011-08-05

**Authors:** Franciele Rosso, Candice T Michelon, Rosa D Sperhacke, Mirela Verza, Liliane Olival, Marcus B Conde, Renata L Guerra, Arnaldo Zaha, Maria LR Rossetti

**Affiliations:** 1 Programa de Pós Graduação em Biologia Celular e Molecular - Universidade Federal do Rio Grande do Sul - Av. Bento Gonçalves, 9500, Cx. Postal 15005, ZIP code: 91501-970 - Porto Alegre, Brazil; 2 Centro de Desenvolvimento Científico e Tecnológico, Fundação Estadual de Produção e Pesquisa em Saúde - Av. Ipiranga, 5400, ZIP code: 90610-000, Porto Alegre, Brazil; 3 Laboratório de Pesquisa em HIV1AIDS, Centro de Ciências da Saúde, Universidade de Caxias do Sul - Rua Francisco Getúlio Vargas, 1130, Bloco S, Sala 315, ZIP code: 95070-560, Caxias do Sul, Brazil; 4 Instituto de Doenças do Tórax, Universidade Federal do Rio de Janeiro - Rua Professor Rodolpho Paulo Rocco, 255, 6° andar, ZIP code: 21941-913, Rio de Janeiro, Brazil; 5 Universidade Luterana do Brasil - Av. Farroupilha, 8001, ZIP code: 92425-900, Canoas, Brazil

## Abstract

**Background:**

Pleural tuberculosis (TB) diagnosis often requires invasive procedures such as pleural biopsy. The aim of this study was to evaluate the role of real-time polymerase chain reaction (PCR) for the *IS*6110 sequence of *M. tuberculosis *in pleural fluid specimens as a rapid and non-invasive test for pleural TB diagnosis.

**Findings:**

For this cross-sectional study, 150 consecutive patients with pleural effusion diagnosed by chest radiography, who were referred for diagnostic thoracocentesis and pleural biopsy and met eligibility criteria, had a pleural fluid specimen submitted for real-time PCR testing. Overall, 98 patients had pleural TB and 52 had pleural effusion secondary to other disease. TB diagnosis was obtained using acid-fast bacilli (AFB) smear or culture for mycobacteria and/or histopathologic examination in 94 cases and by clinical findings in 4 cases. Sensitivity, specificity, positive and negative predictive values of PCR testing for pleural TB diagnosis were 42.8% (95% CI 38.4 - 44.8), 94.2% (95% CI 85.8 - 98.0), 93.3% (95% CI 83.6 - 97.7), and 48.5% (95% CI 44.2 - 50.4), respectively. The real-time PCR test improved TB detection from 30.6% to 42.9% when compared to AFB smear and culture methods performed on pleural fluid specimens, although the best sensitivity was achieved by combining the results of culture and histopathology of pleural tissue specimens.

**Conclusion:**

The real-time PCR test of pleural fluid specimens is a useful and non-invasive additional assay for fast diagnosis of pleural TB.

## Introduction

Diagnosis of pleural tuberculosis (TB) remains a challenge due to its nonspecific clinical presentation and paucibacillary nature. Conventional methods, such as direct testing for acid-fast bacilli (AFB) and culture of pleural fluid, lack sensitivity (less than 5% and 40%, respectively) [1-3]. Despite improved detection rates with new methods, high pleural fluid levels of adenosine deaminase (ADA) may be found in other diseases, especially empyema and parapneumonic effusions, and there is no established cut-off value for measurement of interferon-gamma in pleural fluid [4,5].

Therefore, a definitive diagnosis of pleural TB still depends on demonstration of *M*. *tuberculosis *or caseous granulomas in pleural biopsy, an invasive method with possible complications requiring specialized medical care that is not widely available [6]. Nucleic acid amplification assays allow the direct detection of *M. tuberculosis *in clinical specimens within hours of their receipt in the laboratory, providing a potential tool for diagnosing paucibacillary forms of TB [7]. Although the role of nucleic acid amplification tests has been reasonably well defined in pulmonary tuberculosis, their place in pleurisy evaluation is not clear [7,8]. Real-time polymerase chain reaction (PCR) is a technology that has advantages over conventional PCR testing, such as the fast availability of results, decreased risk of contamination and quantification of bacterial load [9].

In Brazil, TB has an initial presentation as a pleural disease in about 9% of adults and is the main cause of pleural effusion, corresponding to over 50% of cases [10,11]. In this study, we evaluated the role of a real-time PCR assay for the *IS*6110 sequence of *M. tuberculosis *in pleural fluid specimens to evaluate the efficiency and practicality of the test for diagnosing pleural TB.

## Materials and methods

### Study population

From January 2003 to July 2005, all patients presenting with pleural effusion on chest radiography referred by their clinicians to the Pulmonary Service for Diagnostic Methods, Thoracic Diseases Institute/Clementino Fraga Filho University Hospital Federal University of Rio de Janeiro for diagnostic thoracocentesis were eligible for the study. Inclusion criteria were age ≥ 18 years old, absence of known history or clinical or radiographic evidence of renal, cardiac or liver failure, and no known diagnosis of cancer or TB at the moment of enrollment. All included patients signed a consent form and were submitted to an interview, physical examination, and thoracocentesis followed by pleural biopsy with Cope's needle. Patients were excluded from the study if the diagnostic procedures were not completed or if the pleural effusion etiology remained undetermined at the end of the diagnostic investigation. Pleural effusion was deemed limited if it affected less than one third of the hemithorax, moderate if it affected between one third and two thirds of the hemithorax, and extensive if it affected more than two thirds of the hemithorax. This study was approved by the Research Ethics Committees of the Federal University of Rio de Janeiro and of the State Foundation of Health Research and Production of Porto Alegre, Brazil.

### Clinical specimens

Two pleural biopsy fragments and an aliquot of pleural fluid were stained using the Ziehl-Neelsen method and cultured in Löwenstein Jensen and Sabouraud medium following standard protocols [12]. All specimens with positive mycobacteria culture results were tested using biochemical methods to distinguish *M. tuberculosis *from other nontuberculous mycobacteria. Three pleural biopsy fragments were submitted to histopathological examination after hematoxylin and eosin staining, and an aliquot of pleural fluid was stained using the Papanicolaou method. Immediately after pleural fluid collection, an aliquot of 500 μL was frozen at -20°C and sent to the State Foundation of Health Research and Production of Porto Alegre for real-time PCR testing.

### Pleural TB definition

A diagnosis of pleural TB was made using the following tests: AFB smear, the growth of *M. tuberculosis *(MTB) in pleural fluid or pleural biopsy tissue, the presence of granulomatous inflammation in histopathologic study of the pleural biopsy tissue, or the resolution of clinical and radiograph abnormalities after six months of standard antituberculosis treatment (clinical diagnosis).

### DNA extraction

DNA was extracted from clinical specimens according to previously described methodology with modifications [13]. Briefly, pleural fluid samples were concentrated by centrifugation, and 100 μL of lysis buffer (8 M guanidine hydrochloride, 0.08 M Tris HCl, 0.04 M EDTA and 2% Triton X-100) was added to the pellet and vortexed. Lysed samples were boiled for 10 min and centrifuged, and the resultant supernatants were transferred to a tube containing 2.5 μL of silica suspension. After centrifugation, the supernatant was removed, and the pellet was washed twice with washing buffer (8 M guanidine hydrochloride and 0.08 M Tris HCl) and once with 70% ethanol. The tubes were dried with closed lids at 56°C for 10 min and with open lids at room temperature for 5 min. Elution buffer (1× TE) was added, and the tubes were homogenized and incubated for 10 min at 56°C. After centrifugation, the supernatant was collected for further experimentation. An extraction negative control (tube containing no organisms) and an extraction positive control (dilution of a commercial *M. tuberculosis *H37Rv reference strain ATCC27294 containing 50 colony forming units) were processed in each set of DNA extractions.

### Real-time PCR assay

Amplification reactions were performed with primers INS-1/INS-2 as previously described, which generated a 245-base pair (bp) amplification product [14]. PCR was performed using the ABI Prism 7500 system (Applied Biosystems, Foster City, California), and the amplified product was detected with SYBR Green I dye. The reaction was optimized to obtain the best amplification kinetics. The final reaction volume (20 μL) contained a mixture of 5 μL of extracted target DNA, 10 μL (0.8 ×) of SYBR Green PCR Master Mix (Applied Biosystems), primers INS-1/INS-2 (5 pmol) and water. After 2 min of incubation at 50°C and 10 min at 95°C, 40 cycles of amplification were performed with a thermal profile of 95°C for 15 sec and 68 °C for 1 min. A final cycle (95°C for 15 sec, 60°C for 1 min, 95°C for 15 sec) was added for the dissociation curve. The amplification and dissociation curves were analyzed with 7500 System Software (Applied Biosystems) with threshold and baseline analysis autodefined by the software. A negative control (master mix with water instead of template DNA) and a positive control (pAMP-1 plasmid containing an *IS*6110 245-bp inserted fragment) were included for each set of PCRs. The sample was defined as positive when it had a detectable cycle threshold (Ct) and the melting temperature (Tm) was the same as for the positive control (88.8°C). Our group has obtained successful results using the *IS*6110 region to detect *M. tuberculosis *complex DNA, and thus this region was chosen for the study [15,16].

### Presence of inhibitors

For all specimens with negative results, an internal control (pAMP-1 plasmid containing an exogenous 600-bp fragment obtained from the pUC13 plasmid digested with *Eco*RI enzyme) was added to the PCR (10 fg/μL, the detection limit concentration) to evaluate the presence of inhibitors. This sequence was amplified as described by Cortez-Herrera et al [17] using the same primers INS-1/INS-2 to generate a 664 bp amplicon. PCR products were analyzed using electrophoresis on a 1.5% agarose gel stained with ethidium bromide and visualized with an ultra-violet illuminator to allow the identification of the amplicon size [14].

### Statistical analysis

The Chi-squared test was used to compare frequencies between groups, and Fisher's exact test was used when the absolute number compared was less than 5. Sensitivity, specificity, positive predictive value (PPV), negative predictive value (NPV) and 95% confidence interval (CI) were calculated. Analysis was performed using SPSS 12.0 software (SPSS Ins. Chicago, IL, USA).

## Results

A total of 158 patients were enrolled in the study. Eight subjects were excluded because they did not complete all diagnostic procedures. Among the remaining 150 patients, 98 (65.3%) had pleural TB, and 52 (34.7%) had pleural effusion secondary to other disease (33 neoplasia, 8 unspecified inflammatory disease, 7 pneumonia, 3 edemigenic syndrome, and 1 systemic lupus erythematosus). Among the patients with TB diagnosis, all had a negative HIV test. A cough was described in 69.4% (68/98), thoracic pain in 75.5% (74/98), fever in 82.6% (81/98) and dyspnea in 20.4% (20/98) of the TB patients. Demographic characteristics of the patients are presented in Table 1.

**Table 1:** Demographic characteristics of 150 patients with pleural effusion.

	TB (n = 98)	non-TB(n = 52)	p value
**Mean age (years)**	36	60	<0.01
**Gender [n (%)]**			0.40
Male	67 (68.4)	32 (61.5)	
Female	31 (31.6)	20 (38.5)	
**Race [n (%)]**			0.12
White	45 (46.4)	31 (59.6)	
Non-white	52 (53.6)	21 (40.4)	
Unknown	1	0	
Pleural effusion extension*^&^[n (%)]			0.03
Limited	25 (25.8)	18 (35.3)	
Moderate	59 (60.8)	20 (39.2)	
Extensive	13 (13.4)	13 (25.5)	
Unknown	1	1	
Pleural effusion location* [n (%)]			0.63
Unilateral	93 (95.9)		48 (94.1)
Bilateral	4 (4.1)	3 (5.9)	
Unknown	1	1	
Parenchyma alteration* [n (%)]			0.22
No	65 (67)	29 (56.9)	
Yes	32 (33)	22 (43.1)	
Unknown	1	1	

* Chest radiographic aspects

& Limited: pleural effusion affecting less than one third of the hemithorax

Moderate: pleural effusion affecting between one third and two thirds of the hemithorax Extensive: pleural effusion affecting more than two thirds of the hemithorax.

Among the pleural TB cases, 4 (4%) were diagnosed based on the resolution of clinical and radiograph abnormalities after six months of standard anti-TB treatment, and 94 (96%) had diagnosis confirmed by laboratory methods. The most frequent finding was the demonstration of granuloma in pleural biopsy (88.8%; 87/98 patients). AFB smear in pleural fluid and pleura tissue specimens contributed one case each (1%). Both also had cultures positive for MTB. The culture was positive in pleural fluid and pleural tissue specimens in 30.6% (30/98) and 64.3% (63/98) of the patients, respectively.

The accuracy of the PCR test for pleural TB diagnosis is shown in Table 2. Sensitivity and negative predictive value (NPV) were 42.8% (42/98) and 48.5% (49/101), respectively, while specificity and positive predictive value (PPV) were 94.2% (49/52) and 93.3% (42/45), respectively. Pleural fluid obtained from four TB patients repeatedly demonstrated inhibition in the amplification reaction; therefore, inhibition results were only included in the denominator for test accuracy calculations. Three of the four TB patients with inhibited PCR results in pleural fluid (75%) were male, white and presented with unilateral pleural effusion without associated pulmonary parenchyma alteration on chest radiography. All of them had positive cultures from pleural fluid specimens and demonstration of granuloma in pleural biopsy.

**Table 2:** Accuracy of real-time PCR testing in pleural fluid for TB diagnosis.

PCR	TB	non-TB	Sens (%) (95% CI)	Spec (%) (95% CI)	PPV (%) (95% CI)	NPV (%) (95% CI)
Positive (n = 45)	42	3	42.8		93.3	
Negative (n = 101)	52	49	(38.4 - 44.8)	94.2	(83.6 - 97.7)	48.5
Inhibited (n = 4)	4	0		(85.8 - 98.0)		(44.2 - 50.4)
Total (n = 150)	98	52				

PCR test accuracy was calculated using the inhibition results only in the denominator.

PCR: Polymerase chain-reaction; TB: tuberculosis; Sens: sensitivity; Spec: specificity; PPV: positive predictive value; NPV: negative predictive value; CI: confidence interval

Among the 98 TB patients, pleural fluid analysis provided positive results in a total of 60 patients (61.2%), with 18 specimens positive by culture only, 30 specimens positive by PCR only, and 12 specimens positive in both methods. Therefore, TB was detected in pleural fluid by culture in 30.6% (30/98) of the patients and by PCR testing in 42.9% (42/98) of the patients. Pleural biopsy analysis provided positive results in a total of 90 patients (91.8%), with 3 specimens positive by culture only, 27 positive by histopathology only (presence of granuloma), and 60 specimens positive in both methods. Therefore, TB in pleural tissue was detected by the culture in 64.3% (63/98) of the patients and by histopathologic examination in 88.8% (87/98) of the patients. Table 3 shows the results of real-time PCR, culture for mycobacteria, and histopathology performed in pleural fluid and pleura tissue specimens of the 98 TB patients, including those with solely clinical diagnoses.

**Table 3:** Results of real-time PCR testing, culture for MTB (pleural/tissue specimen), and histopathologic study (presence of granulomatous inflammation in pleural tissue) among 98* patients with pleural TB diagnosis.

	Culture for MTB in pleural fluid		Culture for MTB in pleural tissue		Histopathologic study of pleural tissue		
	positive	negative	positive	negative	positive	negative	Total
Real-time PCR							
positive	12	30	27	15	36	6	42 (42.9%)
negative/inhibition	18	38	36	20	51	5	
Total	30 (30.6%)		63 (64.3%)		87 (88.8%)		98 (100%)

PCR: Polymerase chain-reaction. MTB: *Mycobacterium tuberculosis*. *Four patients with pleural TB had a diagnosis based only on clinical criteria, and three of them had positive PCR results.

## Discussion

Molecular methods have been shown to be important tools for confirming pleural TB diagnosis because of their high specificity (>90%), but they are non-invasive and low risk, unlike the gold-standard method of pleural biopsy [8]. Nevertheless, the sensitivity of PCR testing has been varied, ranging from 37% - 77% across studies, mainly due to different target nucleic acid sequences and the amplification methods used [8]. Low sensitivity values can be explained by the low bacillary load and the presence of substances that inhibit amplification in pleural fluid [18]. Some studies comparing conventional PCR methods with real-time PCR testing have demonstrated higher sensitivities for the latter (difference of about 30% with real-time PCR sensitivity up to 68%) when both tests are performed in the same specimens, especially in pleural biopsies [19,20]. In addition, real-time PCR has the advantages of short turn-around time, lower risk of sample contamination, and ability to quantify bacterial load, raising interest for its use in pleural effusion tests [9].

Our study evaluated the real-time PCR testing in pleural fluid, which does not require an invasive procedure for specimen collection, a fact that should be considered in a setting where pleural biopsy is not widely available. Our results demonstrated real-time PCR sensitivity of 42.8% when TB cases are defined based on clinical criteria (with or without laboratory confirmation). Although we have found higher PCR sensitivity than other studies evaluating similar clinical specimens and target nucleic acid sequences for amplification (*IS*6110) [21-24], our results corroborate the suboptimal sensitivity as the main limitation of PCR testing for replacing conventional diagnostic tests for diagnosis of pleural TB. Pleural fluid specimens in 4 patients with positive culture for TB and demonstration of granuloma in pleural biopsy repeatedly demonstrated inhibition of the nucleic acid amplification reaction. Nonetheless, even without these substances and the 4 false negative results, test sensitivity would not be much improved, from 42.8% to 46.9%. Three false positive samples were observed. This could be potentially due to cross-contamination that could have occurred during specimen processing, although the guidelines for diagnostic methods were followed. The guidelines include unidirectional work flow comprising the physical separation of specimen processing and reagent preparation and the use of positive and negative controls during DNA extraction and PCR amplification [16,25].

Real-time PCR of pleural fluid was able to detect TB in 30 patients with negative AFB smears and culture in pleural fluid and in 3 of 4 patients with pleural TB diagnosed based on clinical criteria alone. Therefore, PCR testing was responsible for an improvement in TB diagnosis from 30.6% to 42.9% when compared to AFB smear and culture methods performed on pleural fluid specimens. In addition, the combined results of culture and PCR in pleural fluid specimens provided an even better rate of pleural TB detection of 61.2% (60/98). Although the best sensitivity was achieved by combining the results of pleura tissue culture and histopathology (91.8%, 90/98), the prompt confirmation of tuberculous pleuritis in about 43% of the patients using the PCR test would allow early treatment initiation. Rapid diagnosis and initiation of chemotherapy are essential to prevent secondary fibrothorax and to avoid subsequent pulmonary or extrapulmonary TB development [5].

Therefore, a negative PCR result does not exclude the diagnosis of pleural TB, as shown in our study, with approximately 50% of the patents with a negative PCR result had a pleural TB diagnosis. Thus, the role of real-time PCR in pleural fluid specimens of patients suspected of having TB would be to rapidly confirm the disease without waiting for culture and histopathologic results. Especially in settings with high TB prevalence, real-time PCR would provide substantial savings for both the patient and the health care provider in terms of an earlier definitive diagnosis, optimum patient care, improved outcomes, and reduced health-care costs [7].

## Conclusion

A real-time PCR assay from pleural fluid specimen is a useful and non-invasive additional test for fast diagnosis of pleural TB.

## Competing interests

The authors declare that they have no competing interests.

## Authors' contributions

FR: participated in the conception and design of the study, collection, analysis and interpretation of data, statistical analysis and drafted the manuscript. CTM: participated in the design of the study and drafted the manuscript. RDS: participated in the conception and design of the study, analysis and interpretation of data. MV: participated in the design of the study and drafted the manuscript. LO: participated in the design of the study and statistical analysis. MBC: helped in the acquisition of funding, participated in the analysis and interpretation of the data and revised the manuscript. RLG: participated in statistical analysis, interpretation of data and drafted the manuscript. AZ: drafted and revised the manuscript. MLRR: helped in the acquisition of funding, participated in analysis and interpretation of data and revised the manuscript. Participated in the general supervision of the research group. All authors read and approved the final manuscript.
